# Adolescent suicide: an individual disaster, but a systemic failure

**DOI:** 10.1007/s00787-021-01834-2

**Published:** 2021-07-01

**Authors:** Marialuisa Cavelti, Michael Kaess

**Affiliations:** 1 University Hospital of Child and Adolescent Psychiatry and Psychotherapy, University of Bern, Bern, Switzerland; 2 Department of Child and Adolescent Psychiatry, Centre for Psychosocial Medicine, University Hospital Heidelberg, Heidelberg, Germany

Suicide is among the leading causes of death worldwide, especially among adolescents [8, 24]. The estimated suicide rate for 10- to 19-year-olds based on WHO mortality data from 2010 to 2016 is 3.77/100,000 people [12]. Estimated lifetime prevalence of suicide ideation, plans, and attempts among adolescents are 12.1%, 4.0%, and 4.1%, respectively [20]. The risk of suicide is relatively low in childhood, and significantly increases from early adolescence to late adolescence and early adulthood [7, 12]. The high prevalence and significance of adolescent suicidal behavior stand in strong contrast to the fact that “at-risk youths” are notoriously poor help-seekers [30]. For example, a recent study in adolescents between 11 and 19 years of age found a delay of approximately one year between the first suicide attempt and receiving appropriate professional care [19].

Since adolescence represents a critical time window of opportunity for early detection, prevention and intervention of suicidal behavior, great efforts have recently been made to identify risk and protective factors for suicidal behavior in adolescence [2, 7, 29]. The knowledge of risk and protective factors paves the way for new efforts to identify those adolescents who are at risk for suicide, and for indicated intervention targeting this group, along with population-based universal suicide prevention strategies. In this editorial, we highlight interpersonal risk and protective factors for suicidal behavior in adolescents, and call for a more systemic approach in prevention and treatment programs.

In this issue of *European Child and Adolescent Psychiatry*, Grande and colleagues [13] reported a range of family characteristics associated with the occurrence of adolescent suicide. Indeed, living in an atypical family constellation [13, 28], parental separation or divorce, unemployment or low income [23]), parental or family history of mental illness [25] or parent loss due to suicide or death due to another cause [5, 6] range among the most common familial risk factors for adolescent suicide and suicidal behavior. In addition, interpersonal risk factors, such as the quality of relationships to parents, peers, and teachers, seem to play an important role in the etiology of suicidal behavior in adolescents. For example, early maltreatment (e.g., emotional or physical neglect and emotional, physical, or sexual abuse) is a well-established risk factor for suicidal behavior in adolescents (for systematic reviews and meta-analyses, see [1, 27, 35]. The timing of maltreatment seems to matter, with earlier exposure having an even more devastating impact on the young person compared to later exposure [1, 9]. This finding may be partly explained by the fact that the risk for suicidal behavior is higher when the abuser is a member of the immediate or extended family compared to a non-related person [3]. Maltreatment does not necessarily have to happen within family relationships, but the likelihood of that happening seems to be higher the younger the child is. Besides maltreatment, other aspects of family relationships have also been linked to suicide risk in youth, such as negative family climate, family conflict, or maladaptive parenting (e.g., low parental monitoring or inconsistent discipline) [18, 27]. In contrast, parental support, family cohesion, and a consistent discipline by parents have been identified as protective factors for suicidal behavior in adolescents [17, 26, 32].

While at the beginning of life relationship experiences primarily occur within familial interactions, it is one of the developmental tasks of adolescents to gradually take a step back from the family and to increasingly build up peer and other social relationships. Accordingly, it may be little surprising that there is evidence for bullying victimization, a lack of close friends or low peer network integration and cohesion being risk factors for suicidal behavior in youth [4, 16, 17, 31, 34]. At the same time, evidence also suggests that social and school connectedness or support by peers and teachers can mitigate the suicidal risk in teens [10, 15]. Taken together, with the transition from childhood to adolescence, peers and the school context become a source of both, risk and support.

In sum, interpersonal risk factors range among the most important and robust for adolescent suicide and suicidal behavior. Thus, it seems justified to regard suicide as a systemic failure, meaning that the sum of an adolescent’s interpersonal relationships did finally not meet the individual’s needs, which may of course be additionally increased by individual vulnerability and risk factors (e.g., psychiatric disorders, substance misuse or personality traits such as impulsivity).

The knowledge of potential risk factors for suicidal behavior in adolescents enables early identification, but identification alone is not effective in reducing suicidal risk; it needs to be followed by treatment addressing both the identified individual and systemic risk factors. Recent systematic reviews and meta-analyses provide evidence that there are a few effective treatments reducing suicidal behavior in adolescents [11, 14, 22]. Effective interventions have in common that they combine an individual skills training (e.g., emotion regulation, distress tolerance, mindfulness, interpersonal effectiveness, problem-solving) with an active family therapy or parent training component (e.g., parent/family psychoeducation, emotion regulation skills, communication skills, problem-solving skills). This finding may not seem surprising if we consider the above-summarized risk and protective factors within family relationships for adolescent suicidal behavior. Thus, effective treatments may have the power to reverse familial risk mechanisms as well as to improve the quality of the parent–child relationship, and consequently act as a buffer against the negative impact of interpersonal life stressors on the suicidal risk in young people. In addition, school-based prevention, which has shown to be an effective method of suicide prevention (e.g., [33]), may set a stronger focus on school climate and peer relationships. As an example, reduction of bullying via so-called whole school approaches (e.g., [21]) may also reduce suicide risk in adolescents.

In conclusion, research on adolescent suicide over the last decades has revealed that relationship within families and the wider social context (e.g., relationships with peers or teachers) can represent both a source of risk as well as a source of protection and support that fosters resilience. Thus, future universal prevention may set a stronger focus on improving interpersonal relationships (e.g., by improving parenting competencies or by bullying prevention). Additionally, indicated interventions for adolescents with a history of suicidal behavior that target socially driven processes (e.g., family or social support) along with individual (cognitive-behavioral and self-regulatory) processes seem to be most effective in diminishing the suicidal risk in adolescents. The global health problem of adolescent suicide means not only an individual disaster, but also a systemic failure. If we are genuinely interested in providing better support for our youth at suicidal risk, a more systemic approach to risk assessment and suicide intervention and prevention is warranted.
